# Calu-3 cells as novel efficient cell culture system for Borna disease virus 1 (BoDV-1)

**DOI:** 10.1016/j.virusres.2026.199795

**Published:** 2026-09-10

**Authors:** Lisa Arnold, Vanessa Schwarz, Gertrud Knoll, André Gessner, Barbara Schmidt, Markus Bauswein

**Affiliations:** a Institute of Medical Microbiology and Hygiene, University of Regensburg, Regensburg, Germany; b Institute of Clinical Microbiology and Hygiene, University Hospital Regensburg, Regensburg, Germany

**Keywords:** Borna disease virus 1 (BoDV-1), Replication, Cell culture, Virus stock, Calu-3, Vero, Favipiravir (T-705)

## Abstract

Borna disease virus 1 (BoDV-1) is the causative agent of zoonotic infections resulting in severe encephalitis with a high case-fatality rate in humans. To search for an efficient *in-vitro* cell culture system for the generation of BoDV-1 virus stocks, we compared different human cell lines (A549, Caco-2, Calu-3, oligodendroglioma cells, SK-N-AS) and Vero cells with respect to BoDV-1 infection kinetics. While all cell lines included in the study supported the release of BoDV-1 RNA and its intracellular accumulation at the end of the experiment, viral RNA was detected in the supernatant of Calu-3 cells as early as six days post infection. In addition, two weeks post infection RNA loads remained highest in the supernatant of this cell line, which also yielded highest BoDV-1 RNA copies per cell. Using the ratio of BoDV-1 RNA in the supernatant and the cell pellet as a surrogate marker, Calu-3 cells released BoDV-1 RNA most efficiently. Notably, favipiravir (T-705), a drug with a described cell-type dependent inhibitory activity against BoDV-1 *in vitro*, which has been used as an off-label therapy in some cases of human BoDV-1 infection, did not reduce BoDV-1 RNA shedding into the supernatant of permanently infected Calu-3 cells and had only a limited effect in a *de-novo* infection model. In conclusion, our results underline the broad viral tropism of BoDV-1 *in vitro* and first describe a high release efficiency for BoDV-1 RNA in Calu-3 cells.

## Highlights

• Calu-3 cells were identified as novel cell culture system for Borna disease virus 1.
• BoDV-1 RNA release was most efficient in Calu-3 cells.
• Favipiravir did not inhibit BoDV-1 RNA release in permanently infected Calu-3 cells.

## 1 Introduction

Borna disease virus 1 (BoDV-1) is an enveloped negative-stranded RNA virus (order *Mononegavirales*, family *Bornaviridae*) with a genome of approximately 8.9 kbp (La Torre et al., 1990), which has recently been proven to cause severe zoonotic infections with a high case-fatality rate in humans (Korn et al., 2018; Schlottau et al., 2018). Infections are most likely associated with local spill-over from the reservoir of the bicolored white-toothed shrew (*Crocidura leucodon*) (Niller et al., 2020). While in patients and animal dead-end hosts such as horses and alpacas the virus is highly neurotropic (Vollmuth et al., 2025), the bicolored white-toothed shrew secretes and excretes the virus via saliva, urine, feces, and skin (Nobach et al., 2015). The exact transmission route has not been elucidated, whereas residence in rural endemic regions (southern and eastern Germany) on the fringe of small settlements or in stand-alone locations has been identified as a main risk factor for human infections (Pörtner et al., 2023). The virus is also endemic in Austria, Switzerland, and Liechtenstein, but from these countries no human infections have been reported so far (Bauswein et al., 2025), while until now more than sixty cases of human patients with severe BoDV-1 encephalitis have been notified to German health authorities (Pörtner et al., 2025b). After first unspecific symptoms, BoDV-1 infections lead to severe neurological symptoms such as paresis, ataxia, impaired consciousness, epileptic seizures, and eventually deep coma (Pörtner et al., 2025a). Until now, no therapy has been approved for human BoDV-1 infections. As the virus does not show a cytopathic effect (CPE) in cell culture *in vitro*, a T-cell-driven immunopathology is assumed as important contributing factor of pathogenesis, supported by data from animal experiments (Nobach et al., 2020). Some patients with BoDV-1 infection were treated with immunosuppressive medication (Pörtner et al., 2025a) and in addition favipiravir (T-705) was used as an antiviral agent in some cases (Paal et al., 2025), based on preclinical *in-vitro* data (Tokunaga et al., 2017). Despite individual off-label therapy attempts, case-fatality rates remained high (Grosse et al., 2023). In *post-mortem* tissue samples of patients with BoDV-1 infection, high viral loads were detected in the brain and with lower concentrations also in peripheral nerves (Neumann et al., 2022). As the receptor for viral entry has not been characterized so far, the mechanisms of neurotropism in humans and animal dead-end hosts remain incompletely understood.

The aim of this study was to investigate the viral tropism *in vitro* by comparing different human cell lines (A549, Caco-2, Calu-3, oligodendroglioma (OL) cells, SK-N-AS) and Vero cells with respect to infectability and cell-line-dependent kinetics of viral shedding. In addition, we aimed to identify more effective *in-vitro* cell culture systems for the generation of BoDV-1 virus stocks, as our previously used system of Vero cells does not produce high-titer virus stocks in the supernatant. Possible previously described cell-line-dependent differences in the *in-vitro* efficacy of favipiravir should also be examined, because an antiviral effect has been demonstrated in some cell lines (*e.g.*, Vero cells, OL, 293T cells), but not in the neuroblastoma cell line SH-SY5Y (Teng et al., 2024; Tokunaga et al., 2017).

## 2 Methods

### 2.1 Generation of BoDV-1 virus stocks

The BoDV-1 isolate “Regensburg 2020” was derived from a *post-mortem* human brain sample, initially isolated in Vero cells (see below) (Neumann et al., 2022). The sequence of the isolate was deposited at GenBank (accession number OR468963.1) (Ebinger et al., 2024). Supernatants were harvested, centrifuged at 1000 rcf, and purified through a 0.22 µm pore size PVDF membrane filter (Carl Roth, Karlsruhe, Germany). Aliquots were frozen and stored at −80 °C. RNA copies were quantified by a BoDV-1 RT-qPCR (see below) (Schlottau et al., 2018). For initial experiments, this virus stock generated in Vero cells was used. For further experiments, a higher concentrated virus stock was generated in Calu-3 cells (see below). Calu-3 cells were infected by the initial virus stock generated in Vero cells and supernatants of Calu-3 cells were harvested, centrifuged at 1000 rcf, and purified through a 0.22 µm pore size PVDF membrane filter. Aliquots of the virus stock (BoDV-1 RNA concentrations before freezing: 4.0 × 10^7^/mL) were frozen and stored at −80 °C. From some aliquots of the virus stock, the concentration of infectious viral particles was determined as 1.2 × 10^5^ infectious units/mL (after one freeze-thaw cycle).

### 2.2 Kinetics of BoDV-1 replication in different human cell lines

The BoDV-1 virus stock was used to examine BoDV-1 infection kinetics in different human cell lines (A549, Caco-2, Calu-3, OL, SK-N-AS) and Vero cells.

A549 (RRID:CVCL_0023), Caco-2 (RRID:CVCL_0025), Calu-3 (RRID:CVCL_0609), and Vero cells (RRID:CVCL_0059; originally from the Max-von-Pettenkofer Institute, Munich, Germany) were provided by the Institute of Clinical Microbiology and Hygiene, University Hospital Regensburg, Regensburg, Germany. OL cells (CCLV-RIE_0221) were obtained from the Friedrich Loeffler Institute, Greifswald – Island Riems, Germany. SK-N-AS cells (RRID:CVCL_1700) were a friendly gift of the Department of Neuropathology, University Hospital Regensburg, Regensburg, Germany. For Calu-3 cells, a short tandem repeat (STR) profiling was performed by Microsynth (Microsynth Seqlab, Göttingen, Germany). STR profiling confirmed Calu-3 cells (RRID: CVCL_0609) and showed no detectable contamination with human origin.

One day prior to infection (day −1), 10^4^ cells/well of the respective cell line were seeded in 24-well plates (Sarstedt, Nümbrecht, Germany) in a volume of 200 µL cell culture medium: DMEM (Thermo Fisher Scientific, Waltham, MA, USA) supplemented with 10% v/v heat-inactivated fetal calf serum (FCS), 0.3 mg/mL glutamine, 200 U/mL penicillin, and 90 U/mL streptomycin (all PAN Biotech, Aidenbach, Germany). Medium for SK-N-AS cells was additionally supplemented with 1% v/v MEM Non-Essential Amino Acids Solution (100X) (Gibco, Thermo Fisher Scientific). Cells were incubated in an incubator (Heracell 150i; Thermo Fisher Scientific) at a humidified atmosphere (37 °C; 5% CO_2_). On day 0, cell lines were infected with a multiplicity of infection (MOI) of 0.05, equivalent to 5.0 × 10^2^ infectious viral particles per 10^4^ cells in the final volume of 200 µL. 24 h post infection (p.i.), cells were washed in D-PBS (Thermo Fisher Scientific) twice. Medium was renewed and the volume per well was adjusted to 400 µL. On day 6 p.i. and day 9 p.i, cells were detached with trypsin (PAN Biotech) and all cells were transferred to a 6-well plate (2 mL volume per well; Sarstedt) and a 25 cm^2^ tissue flask (7 mL volume per flask; Sarstedt), respectively.

Supernatants for BoDV-1 RT-qPCR were collected on day 0 (right after infection), day 3 p.i., day 6 p.i. (before detachment with trypsin), day 9 p.i. (before detachment with trypsin), and day 13 p.i. Supernatants were mixed with an equal amount of DLR buffer (0.1 M NaCl, 0.01 M TRIS, 0.5% IGEPAL CA-630 in DEPC H_2_O; pH 7.4) with an RNAse inhibitor (Applied Biosystems, Thermo Fisher Scientific) and were then centrifuged at 1000 rcf.

Cell pellets were harvested on day 14 p.i. A third of the cell pellets was subjected to nucleic acid extraction for further BoDV-1 RT-qPCR, another third was split in a 96-well microtiter plate for immunofluorescence staining (see Supplemental Figure 2), and another third was frozen at —80 °C for further experiments (pooled from three flasks of the same cell line). In addition, a mycoplasma qPCR was performed from extracted nucleic acids of all cell pellets and excluded mycoplasma contamination.

All experiments were performed three times for each cell line (*n* = 3), the kinetics of BoDV-1 RNA release in supernatants (without determination of TCID_50_ in the supernatant and without analysis of the cell pellet) were performed for another six times in Calu-3 cells (altogether *n* = 9 for infection kinetics of Calu-3 cells), because Calu-3 cells were used as control reference when additional cell lines were tested.

The supernatants of day 14 p.i. were centrifuged at 1000 rcf. Supernatants from all three flasks of the same cell line were pooled, additionally purified through a 0.22 µm pore size PVDF membrane filter, frozen, and stored at −80 °C before quantification of infectious viral particles and BoDV-1 RNA copies.

### 2.3 Polymerase chain reactions (PCRs)

#### 2.3.1 Preparation of supernatants

Supernatants from cell cultures were mixed with an equal amount of DLR buffer with an RNAse inhibitor and were then centrifuged at 1000 rcf.

#### 2.3.2 Preparation of cell pellets

A third of each cell pellet from 25cm^2^ tissue culture flasks was suspended in 200 µL inactivation buffer for nucleic acid extraction (guanidine hydrochloride reagent; Roche, Basel, Switzerland). As a control for extraction and inhibition in the BoDV-1 RT-qPCR, samples were spiked with a defined amount of MS2 bacteriophages and an MS2 phage RT-qPCR was performed according to a previously published protocol (Dreier et al., 2005). Total nucleic acids were extracted using the EZ1 Virus Mini Kit v2.0 (Qiagen, Hilden, Germany) on an EZ1 Advanced XL system (Qiagen), according to the manufacturer's instructions. The eluted volume was 120 µL.

#### 2.3.3 PDH PCR for quantification of cell counts

To quantify cell counts in pellets, 5 µL of extracted nucleic acids (from a total eluted volume of 120 µL) were subjected to a pyruvate dehydrogenase (PDH) PCR, which was performed on a StepOnePlus Real-Time PCR System (Applied Biosystems, Thermo Fisher Scientific). The amplification reaction (without reverse transcription to target only genomic DNA) was performed in a total reaction volume of 30 µL using the TaqMan Universal PCR Master Mix (Applied Biosystems, Thermo Fisher Scientific). For human cell lines, the following primers targeting the genetic region of PDH were used, based on published sequences (Koike et al., 1990):

PDH forward primer: 5´-TCG ATC GGG ACT GCT TTC C-3´

PDH reverse primer: 5´-CCC ACA ACC TAG CAC CAA AAG A-3´

PDH probe: FAM-5´-CAT CTC CTT TTG CTT GGC AAA TCT GAT CC-3´-TAMRA

A plasmid standard curve was used for calculating PDH copies from cycle threshold (ct) values. The cell count was calculated from the PDH copies per 5 µL considering the proportion of one-third-of total cell pellets subjected for nucleic acid extraction (factor 3), the proportion of nucleic acids used for PCR in relation to the total eluted volume (factor 24), and the factor of 0.5 to account for PDH copies per cell, assuming two PDH copies in diploid human cells.

For quantification of cells in Vero cell pellets, the following primers and probe targeting the intronic genetic region of PDH subunit alpha were designed with the NIH Primer-BLAST tool, based on the annotated genome (GenBank accession number NC_132933.1) of *Chlorocebus sabaeus* (taxid: 60711):

PDH forward primer: 5´-TGT GAA GGT TAT GTG TTT GGG AAG-3´

PDH reverse primer: 5´-CTA TGT CAG GAC CCT AAG GCT G-3´

PDH probe: FAM-5´-CCA GGG CTG GCC CCC ATG TA´-MBQ-1

As a standard curve, extracted nucleic acids of defined Vero cell counts (10^3^, 10^4^, 10^5^) as determined by staining with 0.4% trypan blue staining solution (Beckman Coulter, Brea, CA, USA) and counting in a Neubauer chamber (Marienfeld, Lauda-Königshofen, Germany) were used.

#### 2.3.4 BoDV-1 mix 1 RT-qPCR

BoDV-1 RT-qPCR was performed according to a previously published protocol (Schlottau et al., 2018). For supernatants, 5 µL of the inactivated supernatant mixed with an equal amount of DLR buffer were subjected to a BoDV-1 RT-qPCR. For cell pellets, 5 µL of the total eluted volume of 120 µL were used for one-step reverse transcription and amplification in a total reaction volume of 30 µL using the TaqPath 1-Step RT-qPCR Master Mix, CG (Applied Biosystems, Thermo Fisher Scientific). BoDV-1 RNA was detected using the mix 1 real-time RT-qPCR assay, targeting the BoDV-1 X/phosphoprotein (P) gene region (Schlottau et al., 2018):

BoDV-1 mix 1 forward primer: 5´-TAG TYA GGA GGC TCA ATG GCA-3´

BoDV-1 mix 1 reverse primer: 5´-GTC CYT CAG GAG CTG GTC-3´

BoDV-1 mix 1 probe: FAM-5´-AAG AAG ATC CCC AGA CAC TAC GAC G-3´-BHQ1

The BoDV-1 mix 1 RT-qPCR was performed on a StepOnePlus Real-Time PCR System. *In-vitro*-transcribed RNA molecules were used as standards for quantification, generated as previously described for a different detection assay (Wenzel et al., 2009). The lower limit of detection (LLOD) of the BoDV-1 mix 1 RT-qPCR was determined as 3.0 × 10^2^ copies/mL.

#### 2.3.5 HAND2 and GAPDH PCR

To measure mRNA expression levels of the cellular protein heart- and neural crest derivatives-expressed protein 2 (HAND2) in SK-N-AS and Calu-3 cells, HAND2 and GAPDH qPCRs were performed on a LightCycler 480 (Roche, Basel, Switzerland) using the SYBR Green I Master (Roche). Extracted nucleic acids from SK-N-AS and Calu-3 cell pellets were reverse transcribed using LunaScript RT SuperMix Kit (New England Biolabs, Ipswich, MA, USA). 2 µL of cDNA were used for qPCRs in a total reaction volume of 20 µL with the following primers (HAND2 primers according to Teng et al. (Teng et al., 2024) and GAPDH primers according to Harmer et al. (Harmer et al., 2002)):

HAND2 forward primer: 5´-CAA AAT CAA GAC CCT GCG CC-3´

HAND2 reverse primer: 5´-GTT GCT GCT CAC TGT GCT TT-3´

GAPDH forward primer: 5´-GAA GGT GAA GGT CGG AGT CAA C-3´

GAPDH reverse primer: 5´-CAG AGT TAA AAG CAG CCC TGG T-3´

For the calculation of mRNA expression levels, the ΔΔCt method was applied.

### 2.4 Quantification of infectious viral particles in supernatants

Infectious viral particles in supernatants were quantified in Calu-3 cells by a qPCR-based-readout endpoint dilution method for TCID_50_-determination according to the basic method of Reed and Muench (Reed and Muench, 1938).

The supernatants of day 14 p.i. were centrifuged at 1000 rcf, were pooled from all three flasks of the same cell line, and were additionally purified through a 0.22 µm pore size PVDF membrane filter, before they were frozen and stored at −80 °C. After thawing, they were subjected to virus quantification as well as BoDV-1 RT-qPCR (for determination of BoDV-1 RNA copies).

Using 48-well plates (Sarstedt), 7.5 × 10^3^ Calu-3 cells per well were seeded in a volume of 200 µL cell culture medium one day before infection (day —1). Supernatants of A549, Caco-2, OL, SK-N-AS, and Vero cells were diluted in a 4-fold dilution series (in cell culture medium), starting with 1:5. Supernatants of Calu-3 cells were diluted in a 10-fold dilution series, starting with 1:20. Supernatants were applied to pre-incubated Calu-3 cells on day 0 in a total volume of 200 µL. Each dilution was performed in six replicates. 24 h after infection, Calu-3 cells were washed in D-PBS twice, medium was renewed, and the volume was adjusted to 400 µL. Supernatants were harvested on day 7 p.i. and on day 15 — 16 p.i. and were subjected to BoDV-1 mix 1 RT-qPCR. Wells with a positive BoDV-1 RT-qPCR signal in the supernatant on day 7 p.i. or day 15 — 16 p.i. were assumed to be “infected”, wells with undetected BoDV-1 RNA in the supernatant were assumed to be “uninfected”. The TCID_50_ per mL supernatant was calculated according to Reed and Muench by cumulating infected (in the corresponding and in all higher dilutions) and uninfected wells (in the corresponding and in all lower dilutions) for each dilution, before the proportional distance between the dilution just over and just under the 50% infection rate was calculated (Reed and Muench, 1938). Infectious units per mL supernatant were calculated from the TCID_50_ per mL by multiplication with a factor of ln (2), assuming a Poisson distribution. The theoretical lower limit of quantification (LLOQ) for infectious particles can be derived assuming that the lowest dilution used in the experiment represents the TCID_50_: ln (2) x 5/mL x 5 × 4° = 17.3/mL for the protocol used for A549, Caco-2, OL, SK-N-AS, and Vero cells ln (2) x 5/mL x 20×10° = 69.3/mL for the protocol used for Calu-3 cells

Supernatants with an accumulated infection rate of the lowest dilution < 50%, for which infected wells were detected, were assumed to contain infectious particles below the LLOQ. Supernatants, for which no infected wells were detected, were assumed not to contain infectious particles.

### 2.5 Favipiravir (T-705)

The effect of favipiravir (T-705) was tested in Vero cells and Calu-3 cells, permanently infected with BoDV-1 (isolate “Regensburg 2020”).

Favipiravir (Sigma-Aldrich/Merck) was diluted in H_2_O to a stock concentration of 10 mM.

For the experiments with permanently BoDV-1-infected Vero or Calu-3 cells, 7.5 × 10^3^ cells per well were seeded in 48-well plates (volume 200 µL) one day before the application of favipiravir (day —1). On day 0, favipiravir was added in the following concentrations: 31.25 µM, 62.50 µM, 125 µM, 250 µM, 500 µM, 1000 µM. Each concentration was tested in triplicate. Cells in medium only served as controls (0 µM favipiravir). For permanently infected Vero cells, one experiment was performed, for permanently infected Calu-3 cells, three experiments were performed. Medium with the corresponding favipiravir concentrations was renewed on day 6 and on day 9 after first application of favipiravir. Supernatants were harvested on 14 after first application of favipiravir and were subjected to BoDV-1 RT-qPCR.

### 2.6 Statistical analysis

Statistical analyses were performed and figures were created using GraphPad Prism, version 10.6.1 (GraphPad Software, San Diego, CA, USA). In the figures geometric means and geometric standard deviations are shown, which are equivalent to means and standard deviations of log_10_-transformed data as exponents to base 10. For statistical analyses, BoDV-1 RNA copy numbers were transformed to log_10_-values. Normal (Gaussian) distribution was assumed for log_10_-transformed data (equivalent to assumed log-normal distribution of untransformed data). The following symbols were used to indicate the level of significance: * (*p* ≤ 0.05); ** (*p* ≤ 0.01); *** (*p* ≤ 0.001); **** (*p* ≤ 0.0001).

## 3 Results

### 3.1 Calu-3 cells efficiently release high concentrations of BoDV-1 RNA in supernatant

To investigate BoDV-1 tropism and kinetics of viral shedding *in vitro*, different human cell lines (A549, Caco-2, Calu-3, OL, SK-N-AS) and Vero cells (for characteristics of cell lines see Table 1) were infected with a BoDV-1 virus stock: The strain “Regensburg 2020” (genetic cluster 4) had been isolated from a human brain sample in Vero cells, before a highly infectious virus stock was generated in Calu-3 cells.

**Table 1:** Characteristics of cell lines.

Cell line	Organism	Tissue	Cell type	Disease
A549	*Homo sapiens*	lung	epithelial cell	lung adenocarcinoma
Caco-2	*Homo sapiens*	large intestine/colon	enterocyte	colorectal adenocarcinoma
Calu-3	*Homo sapiens*	lung	epithelial cell	lung adenocarcinoma
OL	*Homo sapiens*	brain	oligodendrocyte	oligodendroglioma
SK-N-AS	*Homo sapiens*	peripheral nervous system (isolated from a bone marrow metastasis)	neuroblast	neuroblastoma
Vero	*Chlorocebus sabaeus*	kidney	epithelial cell	-

**Fig. 1:**
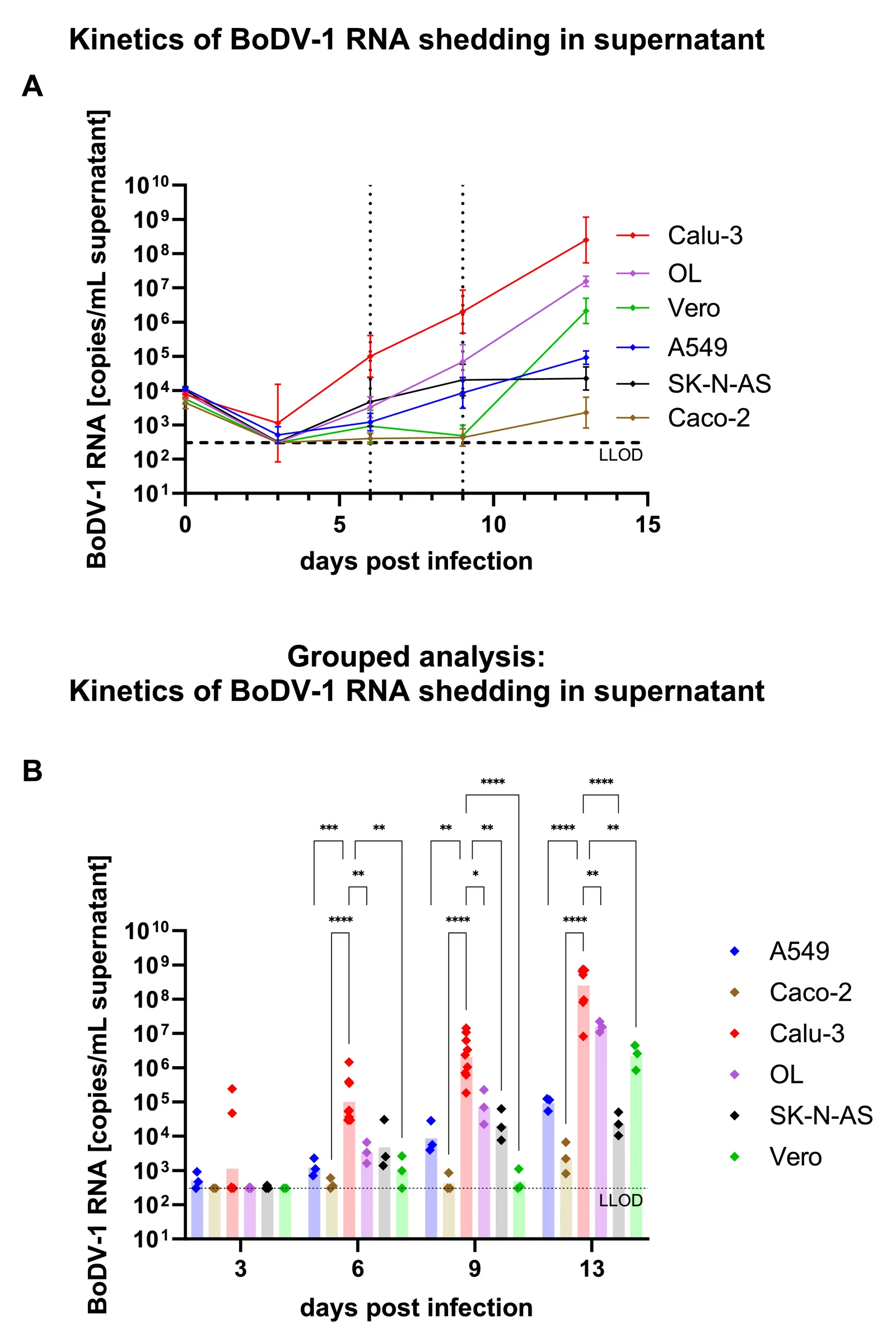
Different human tumor-derived cell lines (A549, Caco-2, Calu-3, OL, SK-N-AS) and Vero cells were infected with a BoDV-1 stock (isolate “Regensburg 2020”, virus stock generated in Calu-3 cells) using a multiplicity of infection (MOI) of 0.05, starting in a 24-well plate. One day post infection (p.i.), cells were washed in D-PBS. Supernatants were collected on day 0, 3, 6, 9, and 13 p.i. and a quantitative BoDV-1 RT-qPCR with the lower limit of detection (LLOD) of 3.0 × 10^2^ copies/mL (indicated by the dotted horizontal lines in **A** and **B**) was performed. When BoDV-1 RNA was undetermined or copies were below the LLOD, a value of 3.0 × 10^2^ copies/mL was assigned. On day 6 p.i. and day 9 p.i., cells were detached with trypsin and all cells were transferred to a 6-well plate and a 25 cm^2^ tissue culture flask, respectively (indicated by vertical dotted lines in **A**). All cell lines showed positive results for BoDV-1 RNA in supernatant on day 13 p.i. RNA copies/mL in supernatant and kinetics varied between cell lines. **(A)** Depicted are geometric means and geometric standard deviations (equivalent to means and standard deviations of log_10_-transformed data expressed as exponents to base 10; *n* = 9 for Calu-3; *n* = 3 for all other cell lines). **(B)** The figure shows individual values and geometric means. A repeated-measures two-way ANOVA with the Geisser-Greenhouse correction was performed with the log_10_-transformed data, followed by Dunnett´s multiple comparisons test with reference to Calu-3 cells. Only statistically significant comparisons are shown. While the unequal sample size between Calu-3 (*n* = 9) and all other cell lines (*n* = 3) does not affect the qualitative result of highest means for Calu-3 cells at every time point ≥ six days p.i. (as it is evident by individual values), statistical significance for some comparisons was only reached with larger sample sizes for Calu-3 cells. Calu-3 cells were always used as reference cell line when additional cell lines were tested sequentially, which is the primary underlying reason for the unequal sample sizes.

In a standardized *in-vitro* infection experiment using this BoDV-1 stock generated in Calu-3 cells, supernatants of Calu-3 cells harbored BoDV-1 RNA copies exceeding the input concentrations of day 0 as early as six days p.i. (Fig. 1). In addition, highest BoDV-1 RNA copies were detected in the supernatants of Calu-3 cells compared to the other cell lines tested at nine days p.i. (geometric mean and geometric standard deviation: 10^6.31±0.59^ BoDV-1 RNA copies/mL; *n* = 9 for Calu-3, *n* = 3 for all other cell lines) and 13 days p.i. (10^8.40±0.64^ BoDV-1 RNA copies/mL; *n* = 9 for Calu-3, *n* = 3 for all other cell lines). While in the supernatants of all cell lines included BoDV-1 RNA was detected on 13 p.i., BoDV-1 geometric mean concentrations exceeded the measured input concentrations of day 0 only in Calu-3, OL, and Vero cells at day 13 p.i.

In conclusion, all cell lines included in the experiment supported detectable release of BoDV-1 RNA in the supernatant. BoDV-1 RNA copies above the input concentration of day 0 were detected in the supernatant of Calu-3 cells as early as six days p.i. and remained highest until 13 days p.i.

A similar phenotype of Calu-3 cells with shedding of high amounts of viral RNA into supernatant had also been observed in initial experiments (*n* = 6) using a virus stock generated in Vero cells (Supplemental Figure S1). Although the experimental conditions (input, splitting, cell transfer) were less standardized for these initial experiments compared to the experiments performed with the virus stock generated in Calu-3 cells, the data clearly show an increased shedding of BoDV-1 RNA by Calu-3 cells in the supernatant, independently of a previous adaption to this cell line.

In addition, similar results of BoDV-1 RNA kinetics in supernatants of Calu-3 cells were obtained in exploratory experiments for the BoDV-1 isolates “Regensburg 2019” (GenBank accession number LR722643.1 (Ebinger et al., 2024)) and “Regensburg 2022” (GenBank accession number OR468968.1 (Ebinger et al., 2024)): Geometric mean concentrations of BoDV-1 RNA in supernatant exceeded input concentrations six days p.i. for Calu-3 cells, but not for A549 or Vero cells (Supplemental Figure S2). Although these experiments were less standardized (no defined MOI), they allow the conclusion that the Calu-3 phenotype is not restricted to a single BoDV-1 isolate and not to certain genetic clusters (isolate 2019: cluster 1A; isolate 2020: cluster 4; isolate 2022: cluster 2).

### 3.2 Higher BoDV-1 RNA copies in Calu-3-cell supernatants are not due to higher cell proliferation

**Fig. 2:**
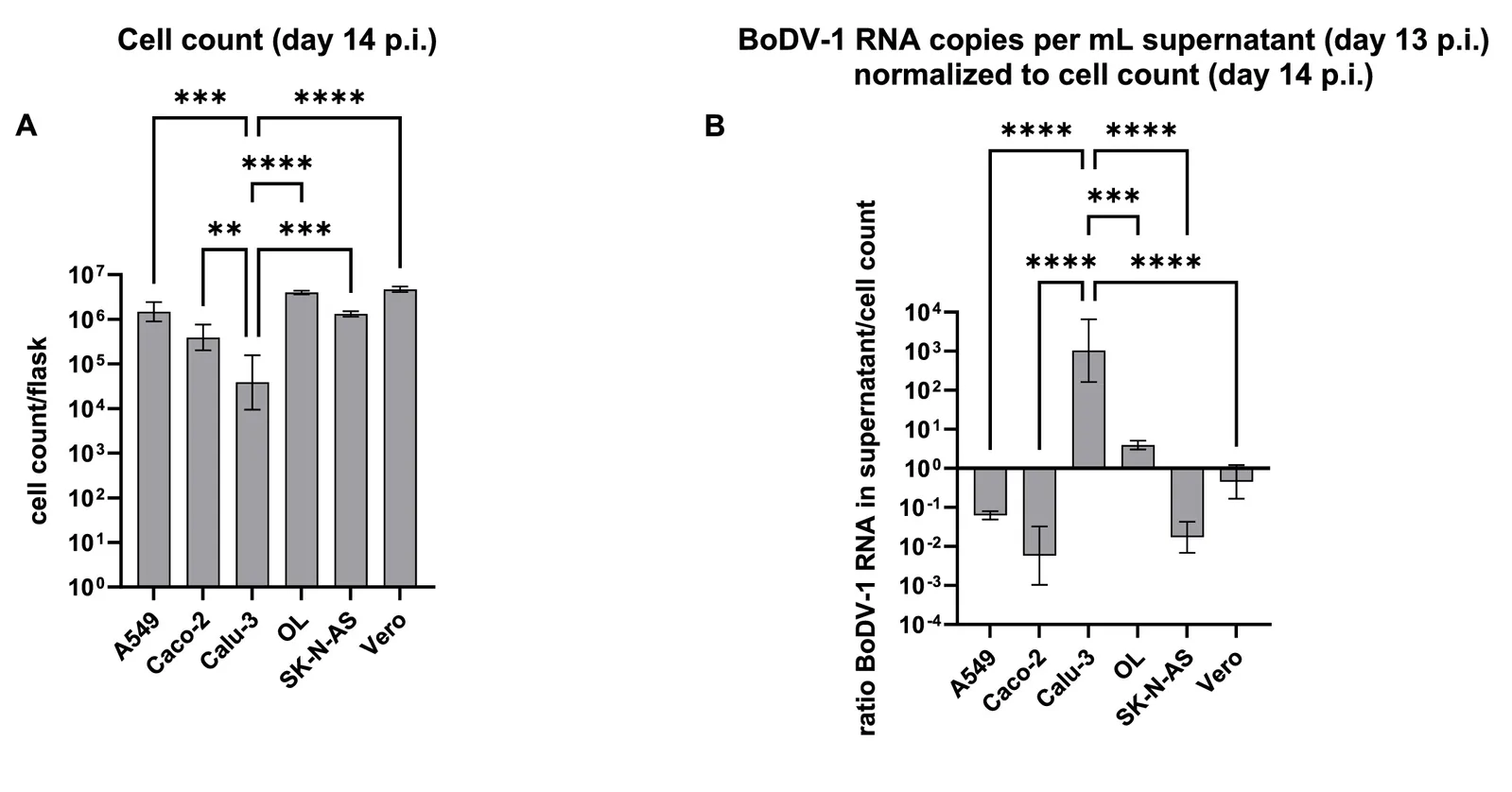
(**A**) Cell counts at the end of the experiment (day 14 p.i.) were approximated by a PCR targeting the pyruvate dehydrogenase (PDH). On day —1 the experiment was started with an equal cell count of 10^4^ for all cell lines. Cells were detached with trypsin and all cells were transferred to 6-well plates and 25 cm^2^ tissue culture flasks on day 6 p.i. and day 9 p.i., respectively. Depicted are geometric means and geometric standard deviations (*n* = 3). For statistical analysis, an ordinary one-way ANOVA followed by Dunnett´s multiple comparisons test with reference to Calu-3 cells was performed with log_10_-transformed data. (**B**) BoDV-1 RNA copies per mL supernatant (day 13 p.i.) were normalized to PDH-PCR-approximated cell counts (day 14 p.i.). Geometric means and geometric standard deviations are depicted (*n* = 3). For statistical analysis, an ordinary one-way ANOVA followed by Dunnett´s multiple comparisons test with reference to Calu-3 cells was performed with log_10_-transformed data.

In a next step, cell pellets harvested on day 14 p.i. (end of the experiment) were analyzed. Total cell counts in cell pellets were approximated by a PCR targeting the genetic region of PDH (Fig. 2A).

While all experiments were started in 24-well plates with an equal cell count of 10^4^ cells on day —1, cell counts approximated by the PDH PCR significantly differed 14 days p.i. As the lowest cell counts were observed for Calu-3 cells, a higher cell proliferation rate and thus a higher total final cell count can be excluded as underlying mechanism for the early and effective shedding of BoDV-1 RNA copies in the supernatants of this cell line compared to the other cell lines. On the contrary, BoDV-1 RNA copies in supernatants (sampled 13 days p.i.) normalized to cell counts (cell count determined 14 days p.i.) were highest for Calu-3 cells (Fig. 2B; *n* = 3).

No cytopathic effect or reduced viability was observed when Calu-3 cell cultures were examined under the microscope. In addition, an LDH release assay was performed with supernatants of Calu-3 and Vero cell cultures permanently infected with BoDV-1 (one experiment with three replicates). Absolute values of LDH activity in the supernatant of infected Calu-3 cells were 259 ± 15 mU/mL (mean and standard deviation) *vs.*150 ± 2 mU/mL in the supernatant of uninfected Calu-3 cells, while in the supernatant of infected Vero cells the absolute LDH activity was measured as 256 ± 27 mU/mL *vs.*197 ± 10 mU/mL in the supernatant of uninfected Vero cells (Supplemental Figure S3). LDH release (expressed as percentage of maximum release of infected cells lysed with Triton X-100) was calculated as 8.9 ± 1.2% (mean and standard deviation) for Calu-3 cells and 2.6 ± 1.2% for Vero cells permanently infected with BoDV-1. Taken together, these data cannot totally exclude a contribution of reduced cell viability for the increased release of BoDV-1 RNA in the supernatant of infected Calu-3 cells, but it seems rather unlikely to be a primary underlying mechanism.

### 3.3 BoDV-1 RNA copies per cell and release of BoDV-1 RNA in supernatant are highest in Calu-3 cells

**Fig. 3:**
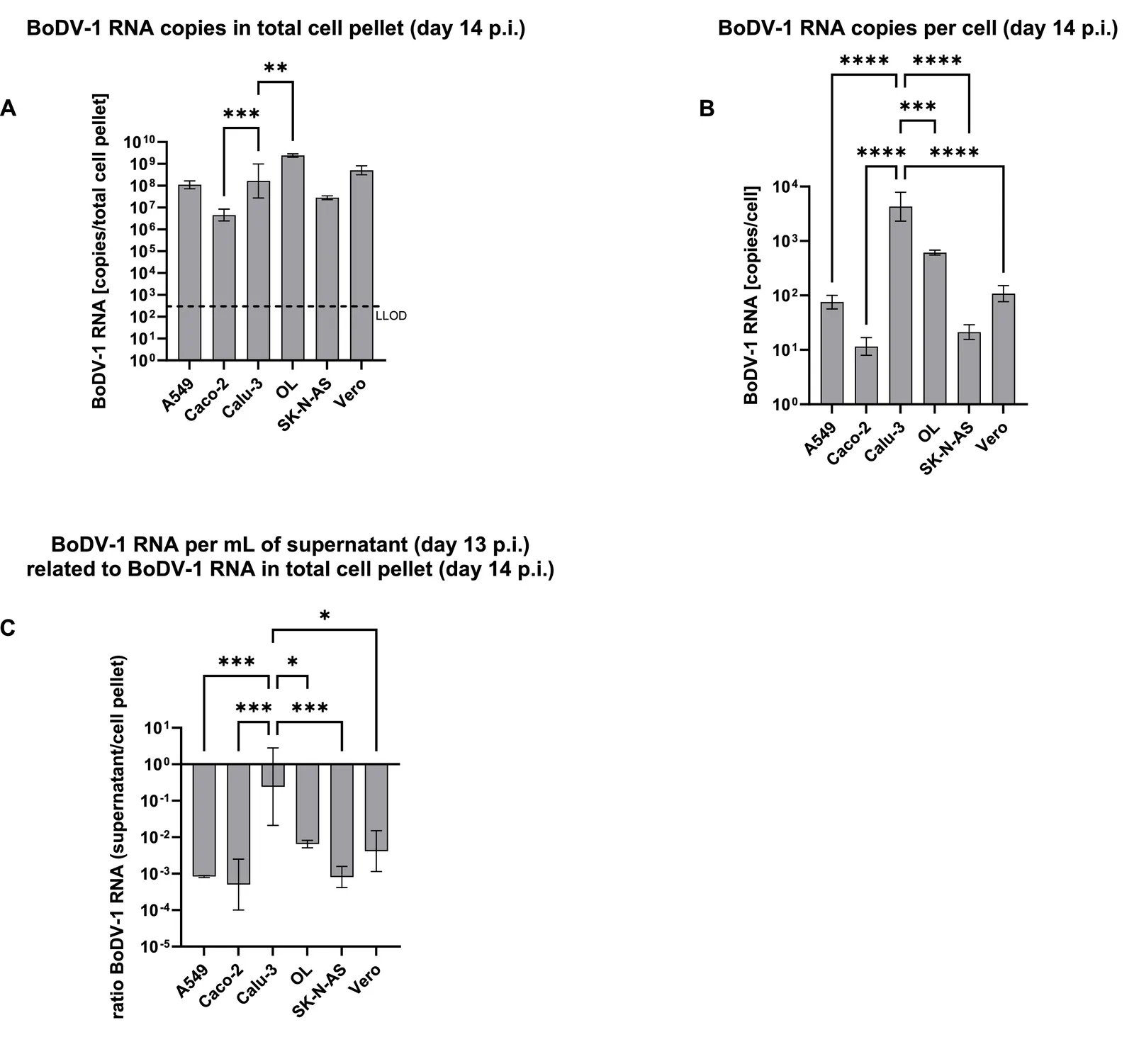
BoDV-1 RNA copies in cell pellets harvested on day 14 p.i. (**A**) shows BoDV-1 RNA copies in total cell pellets of 25 cm^2^ tissue culture flasks. The horizontal line marks the LLOD of the BoDV-1 RT-qPCR. In (**B**) BoDV-1 RNA copies related to one cell are depicted (cell count approximated by PDH PCR). Geometric means and geometric standard deviations are given (*n* = 3). Under (**C**) the calculated ratio of BoDV-1 RNA per mL supernatant (day 13 p.i.) and the BoDV-1 RNA in total cell pellets (day 14 p.i.) is shown (individual values, geometric means, geometric standard deviations; *n* = 3). For statistical analysis, an ordinary one-way ANOVA followed by Dunnett´s multiple comparisons test with reference to Calu-3 cells was performed with log_10_-transformed data. Only statistically significant comparisons are indicated.

In addition, BoDV-1 RNA copies were determined in cell pellets harvested on day 14 p.i. While in total cell pellets, OL cells yielded the highest BoDV-1 RNA copies (Fig. 3A), the highest intracellular BoDV-1 RNA loads normalized to the cell count (normalized by PDH-PCR-approximated cell counts) were observed in Calu-3 cells (Fig. 3B). For Calu-3 cells BoDV-1 RNA copies per one cell were 10^3.63±0.22^ (geometric mean and geometric standard deviation; *n* = 3). An immunostaining (anti-BoDV-1-IgG) was performed with cells 14 days p.i. to estimate the frequency of infected cells (Supplemental Figure S4). High infection rates of Calu-3, OL, and Vero cells were detected. An additional staining (anti-BoDV-1-IgG, DAPI) was performed with frozen cells (frozen 14 days p.i., thawed, taken into culture for another 14 days) (Supplemental Figure S5). Based on the immunostaining (anti-BoDV-1-IgG and DAPI) of cells 28 days p.i., an infection rate of > 90% was estimated for Calu-3, OL, and Vero cells by counting infected cell nuclei (stained by anti-BoDV-1-IgG and DAPI), while only app. 30% of A549, < 10% of Caco-2, and app. 40% of SK-N-AS cells were considered infected. With respect to OL and Vero cells, the higher amount of BoDV-1 RNA copies normalized to one cell in Calu-3 cells is most likely explained by a higher average of BoDV-1 RNA load within one cell of this cell line and not due to differences in the frequency of infected cells.

In a further analysis, the BoDV-1 RNA concentrations per mL of supernatant (of day 13 p.i.) were related to the BoDV-1 RNA amount in total cell pellets (of day 14 p.i.). Using this ratio as a surrogate marker, Calu-3 cells showed the highest BoDV-1 RNA release efficiency compared to all other cell lines (Fig. 3C).

### 3.4 Exploratory determination of viral particles in supernatant

**Fig. 4:**
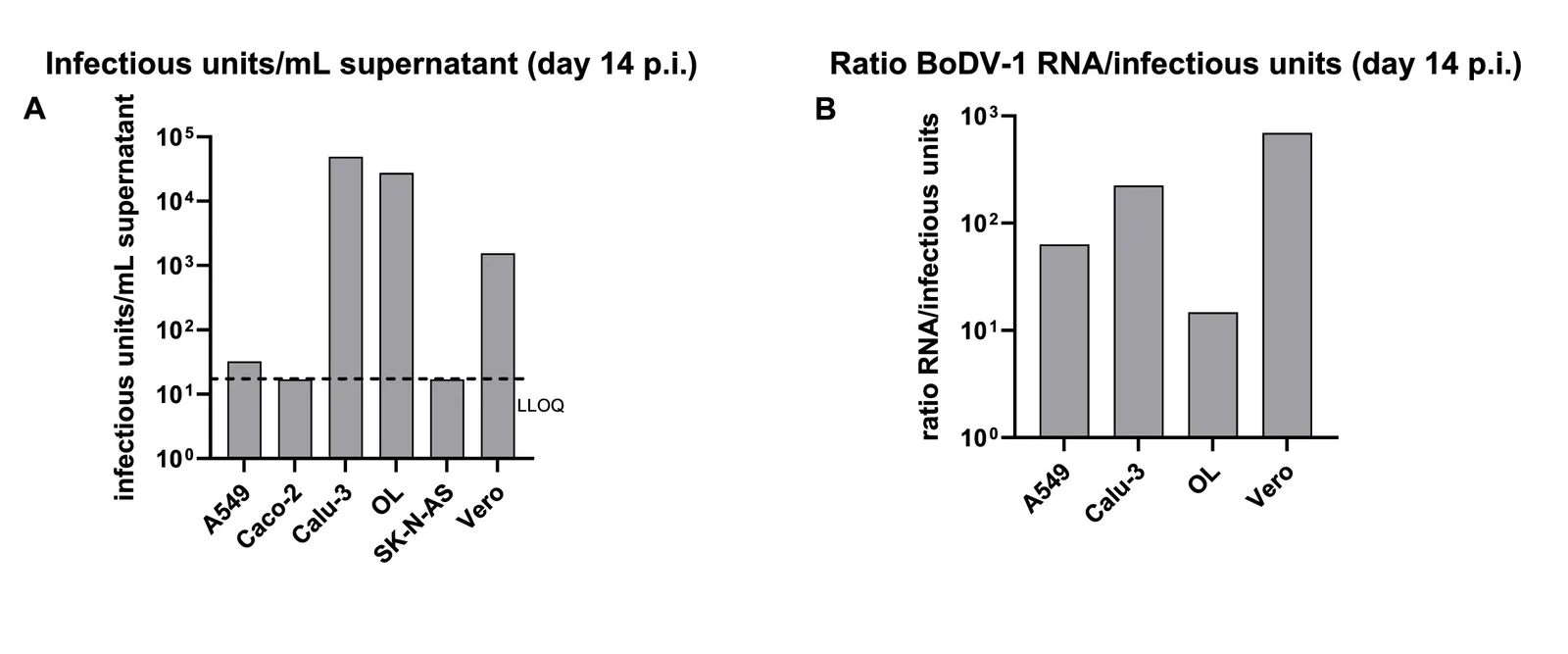
Infectious viral particles in pooled supernatants harvested on day 14 p.i. were quantified by a qPCR-based-readout endpoint dilution assay in Calu-3 cells. The exploratory experiment was performed only once from pooled samples (*n* = 1, pooled supernatants from three experiments). In (**A**), the lower limit of quantification (LLOQ) for the protocol used for A549, Caco-2, OL, SK-N-AS, and Vero cells is represented by the dotted horizontal line. In supernatants of Caco-2 and SK-N-AS cells, infectious viral particles were detected, but they were below the LLOQ. In (**B**), the ratio of BoDV-1 RNA molecules as determined by BoDV-1 mix 1 RT-qPCR to infectious units in supernatant of cells is given.

Infectious units in pooled supernatants of each cell line harvested on day 14 p.i. were estimated by a qPCR-based-readout endpoint dilution assay. Supernatants of three independent experiments were pooled (to obtain sufficient volume), centrifuged, additionally filtrated through a 0.22 µm pore size PVDF membrane filter, frozen, and stored at −80 °C, before infectious viral particles and BoDV-1 RNA copies were determined in an exploratory experiment. Based on one qPCR-based-readout endpoint dilution assay from three pooled supernatants (*n* = 1), Calu-3 cells yielded highest concentrations of infectious viral particle equivalents per mL supernatant compared to the other cell lines (Fig. 4A). For Caco-2, Calu-3, SK-N-AS, and Vero cells BoDV-1 RNA levels in the three independently examined supernatants collected on day 13 p.i. were comparable to BoDV-1 RNA levels in pooled supernatants of day 14 p.i. after additional filtration and one freeze-thaw cycle (difference < 1 log_10_ unit). A significant decrease of BoDV-1 RNA levels, however, was observed for A549 (1.7 log_10_ units), and OL cells (1.6 log_10_ units). OL cells showed the lowest ratio of BoDV-1 RNA copies related to infectious units in the pooled supernatant (Fig. 4B).

### 3.5 Favipiravir (T-705) does not inhibit BoDV-1 RNA shedding into supernatant of permanently infected Calu-3 cells

**Fig. 5:**
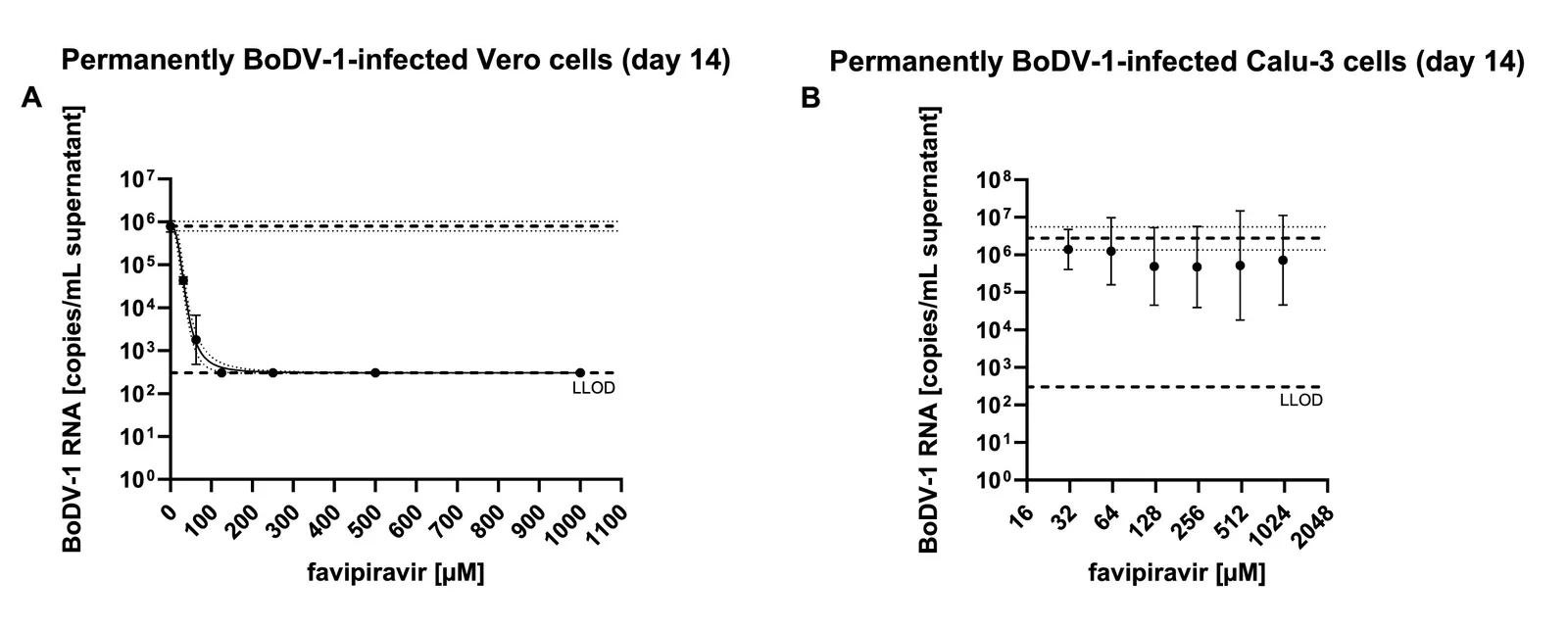
While the known inhibitory effect of favipiravir (T-705) was reproduced in Vero cells (**A**), it did not inhibit the shedding of BoDV-1 RNA in permanently infected Calu-3 cells (**B**). BoDV-1 RNA copies in supernatants of permanently BoDV-1-infected Vero (**A**) and Calu-3 cells (**B**) were determined 14 days after the first application of different concentrations of favipiravir. Geometric means and geometric standard deviations of one (Vero cells) or three experiments (Calu-3 cells) with three replicates each are represented with respect to the y-axis. The lower dotted horizontal lines mark the LLOD of the BoDV-1 RNA RT-qPCR (3.0 × 10^2^ copies/mL). The upper dotted horizontal lines represent geometric means and geometric standard deviations of a control of BoDV-1-infected Vero or Calu-3 cells without favipiravir. Please note the linear x-axis in (**A**) (to include the control with 0 µM favipiravir for a 4PL non-linear regression curve with 95% confidence bands), but the log_2_ x-axis in (**B**).

According to published data in the literature, the drug favipiravir (T-705) showed an inhibitory effect on the replication of BoDV-1 in Vero cells (Tokunaga et al., 2017). This effect was reproduced in our Vero cell line permanently infected with BoDV-1 with an IC_50_ of 38 µM for favipiravir after 14 days of application (one experiment with three replicates) (Fig. 5A), while cell viability was assessed using an MTT assay (Supplemental Figure S6). To compare the Calu-3 cell culture system to the established Vero cell culture system regarding antiviral compound efficacy against BoDV-1, favipiravir was tested in permanently infected Calu-3 cells.

Favipiravir did not reduce BoDV-1 RNA shedding into the supernatant of permanently infected Calu-3 cells under the tested conditions after 14 days of application (*n* = 3 with three replicates each) (Fig. 5B).

In addition, favipiravir was tested in an exploratory *de-novo* infection of Calu-3 cells (Supplemental Figure S7). For the *de-novo* infection of Calu-3 cells, 7.5 × 10^3^ uninfected Calu-3 cells per well were seeded in 48-well plates one day before infection. Favipiravir was added in the following concentrations: 31.25 µM, 62.50 µM, 125 µM, 250 µM, 500 µM, 1000 µM. Supernatants were harvested on day 6, 10, and 14 p.i. and were subjected to BoDV-1 RT-qPCR. Favipiravir did not prevent the *de-novo* qualitative detection of BoDV-1 RNA in the supernatant of Calu-3 cells on day 6, 10, and 14 p.i. While a drop in BoDV-1 RNA copies in supernatants at high concentrations of favipiravir (500 µM and 1000 µM) six days p.i. was not statistically significant, statistical analysis revealed a small (app. 2 log_10_ units) dose-dependent effect of favipiravir on BoDV-1 RNA concentrations in supernatants 14 days p.i. (geometric mean and geometric standard deviation: 10^7.53±0.25^ BoDV-1 RNA copies/mL without favipiravir *vs.* 10^5.49±0.24^ BoDV-1 RNA copies/mL with 1000 µM favipiravir; *n* = 3).

As an interference with the cellular protein HAND2 had been proposed as underlying mechanism for the reduced efficacy of favipiravir in the neuroblastoma cell line SH-SY5Y (Teng et al., 2024), we measured mRNA expression levels of HAND2 in Calu-3 cells in comparison to the neuroblastoma cell line SK-N-AS. Applying the ΔΔCt method with GAPDH as housekeeping gene, we found an app. 10^5^-fold higher expression in uninfected SK-N-AS compared to uninfected Calu-3 cells, in which HAND2 was expressed only very weakly. Furthermore, no change in HAND2 mRNA levels was observed in BoDV-1-infected Calu-3 cells. Thus, interference with HAND2 seems an unlikely explanation for the reduced efficacy of favipiravir in Calu-3 cells.

## 4 Discussion

The aim of this study was to investigate the viral tropism of BoDV-1 *in vitro* by comparing different human cells lines (A549, Caco-2, Calu-3, OL cells, SK-N-AS) and Vero cells regarding infectability and cell-line-dependent kinetics of virus shedding.

All cell lines included in the study supported detectable BoDV-1 RNA release or accumulation under the experimental conditions. No tissue-related restriction was observed as cell lines originating from the lung (A549, Calu-3) and the large intestine/colon (Caco-2) as well as cell lines with origin from the nervous system (OL, SK-N-AS) were susceptible. The broad susceptibility of different cell lines is in line with previous findings from the literature (Teng et al., 2024; Hallensleben and Staeheli, 1999; Tokunaga et al., 2017). Taken together, the available data point to a ubiquitously expressed receptor or to different mechanisms of viral entry. A strong receptor-restriction does not seem to be the underlying cause for neurotropism. The immunological niche within the nervous system and other immunological factors might contribute to the restriction of BoDV-1 to brain and peripheral nerves in humans and other animal dead-end hosts.

Notably, Calu-3 cells released BoDV-1 RNA into the culture supernatant at a relatively early time point (six days p.i.). In addition, BoDV-1 RNA loads in supernatants of this cell line remained highest until the end of the experiment (13/14 days p.i.) compared to the other cell lines tested. Using the ratio of BoDV-1 RNA per mL of supernatant (13 days p.i.) and the BoDV-1 RNA amount in the total cell pellet (14 days p.i.) as a surrogate marker, BoDV-1 RNA release was most efficient in Calu-3 cells. This intriguing finding should be validated in further experiments, for example by freeze-thaw-cycle-induced virus liberation in Calu-3 cells compared to other cell lines.

In a qPCR-based-readout endpoint dilution experiment performed once with pooled supernatants from three experiments, Calu-3 cell supernatants (14 days p.i.) also showed highest infectious units per mL. The ratio of RNA molecules in comparison to estimated infectious units, however, was most favorable in OL cells, assuming that these cells might even be more competent in selectively releasing only mature virus particles than Calu-3 cells. However, this finding should not be overinterpreted, because the qPCR-based-readout endpoint dilution assay was performed only once with pooled supernatants and because an additional filtration step and a freeze-thaw cycle influenced the BoDV-1 RNA load in the OL supernatant more than in the Calu-3 supernatant.

The Calu-3 phenotype with release of high BoDV-1 RNA copies into the supernatant was not only observed in experiments with a virus stock generated in this cell line, but also with the initial virus stock generated in Vero cells. Thus, an adaptation to the Calu-3 cell line as underlying mechanism can be excluded. Furthermore, virus strain-specific effects or a dependence on a specific genetic cluster can be ruled out, since similar phenotypes of BoDV-1 RNA release in the supernatants of Calu-3 cells were also reproduced in exploratory experiments with the BoDV-1 isolates “Regensburg 2019” (cluster 1A) and “Regensburg 2022” (cluster 2), while the initially used BoDV-1 isolate “Regensburg 2020” belongs to the genetic cluster 4.

To our best knowledge, the effective shedding of BoDV-1 RNA in Calu-3 cells has not been described so far. This finding has practical implications: Calu-3 cells allow for time-saving generation of highly concentrated BoDV-1 virus stocks as it has already been done for the virus stock used in this study. In addition, they might simplify tests for neutralizing antibodies by shortening incubation times.

Further, one might speculate, if the observation of the effective BoDV-1 RNA release from Calu-3 cells could potentially point to a role of the upper respiratory epithelium in viral entry. Calu-3 cells are a widely-used model for upper-respiratory-tract infections (Haghi et al., 2010), although they are derived from a lung adenocarcinoma and do not necessarily represent normal nasal epithelium. Compared to A549 cells, another cell line originating from a lung adenocarcinoma, Calu-3 cells were much more efficient in shedding BoDV-1 RNA. As one possible entry site for BoDV-1 infections in humans and other animal dead-end hosts, the nasal route via the olfactory nerve has been described (Kupke et al., 2019). Kupke et al. studied different cell types involved in the intranasal BoDV-1 infection of adult rats *in vivo* and *in vitro*. Interestingly, they detected viral antigen and viral mRNA not only in olfactory receptor neurons, but also in all other cell types of the olfactory mucosa, *in vivo* and *in vitro*, and concluded that olfactory ensheathing cells might play a crucial role for infections via the olfactory route. Whether there is local viral replication of BoDV-1 in neighboring mucosal or olfactory ensheathing cells in humans, which enables the uptake in nerval endings of the olfactory nerve, remains to be elucidated.

Notably, favipiravir did not inhibit the release of BoDV-1 RNA in the supernatant of Calu-3 cells under the tested conditions in permanently infected Calu-3 cells. In a *de-novo* infection model, favipiravir did not prevent the *de-novo* infection of Calu-3 cells, while a minor (app. 2 log_10_ units) dose-dependent decrease of BoDV-1 RNA concentrations in supernatants was observed 14 days p.i.

In Vero, OL, and 293T cells an inhibitory effect of favipiravir against BoDV-1 and other bornaviruses has been shown (Teng et al., 2024; Tokunaga et al., 2017). In our study, we reproduced the effect of favipiravir in Vero cells. The IC_50_ determined in our experiment for viral shedding into the supernatant was lower than the one reported in the literature for other experimental settings (Tokunaga et al., 2017). Because our experiment was performed only once (with three replicates), primarily with the aim to show that the used favipiravir was active as expected, our data should not be overinterpreted. A possible explanation for the difference could be the read-out of our experiment that evaluated BoDV-1 RNA copies in the supernatant and not in the cell pellets.

The present study is not the first to demonstrate that favipiravir can fail to inhibit BoDV-1 replication in a cell-line-dependent manner. The cell-line dependency of the *in-vitro* efficacy of favipiravir has already been clearly described before (Teng et al., 2024). However, to our knowledge, a reduced or absent efficacy of favipiravir has previously been reported only for the neuroblastoma cell line SH-SY5Y, in which interference with the cellular protein HAND2 was proposed as underlying mechanism (Teng et al., 2024). For the first time, we report no relevant (permanently infected cells) or only a limited (*de-novo* infection) efficacy in the non-neuronal cell line of Calu-3 cells, in which we found HAND2 expressed only very weakly in comparison to the neuroblastoma cell line SK-N-AS, rendering a similar inhibitory mechanism unlikely. Possible explanations for the observation that favipiravir did not inhibit BoDV-1 RNA release in permanently infected Calu-3 cells could comprise reduced up-take, increased efflux, and interaction with other cellular proteins. A further examination of the cell-line-dependent efficacy of favipiravir, the revelation of its underlying molecular mechanisms, and *in-vivo* studies seem to be urgently necessary to evaluate this drug and its potential for different possible clinical indications such as therapy and post-exposure prophylaxis.

In conclusion, Calu-3 cells appear practically useful for producing BoDV-1-containing supernatants, although the molecular mechanisms underlying this phenotype remain to be determined and should be addressed in further experiments.

## 5 Limitations

The qPCR-based-readout endpoint dilution assay was performed only once with supernatants obtained 14 days p.i. that had been pooled from three experiments (in order to obtain sufficient volume), filtrated, and frozen before analysis. Data of the pooled supernatant do not allow statistical analysis and should not be overinterpreted. Supernatants collected on day 3, 6, 9, and 13 p.i. were only tested by BoDV-1 RTqPCR. PCR data alone do not allow definitive conclusions on the time course of production of infectious viral particles.

Supernatants for qPCR were collected, inactivated by DLR buffer, and then centrifuged. Theoretically, lysis of potential cells in supernatants by DLR buffer and subsequent release of intracellular BoDV-1 RNA into supernatant cannot be excluded. However, BoDV-1 RNA levels in supernatants of Calu-3 cells collected 13 days p.i., that were treated as described, and those of pooled supernatants collected 14 days p.i., that were first centrifuged, filtrated, and frozen, were comparable, rendering this mechanism unlikely as important contributing factor for the observed phenotype of Calu-3 cells.

## Funding

The study was in part funded by the Bavarian State Ministry of Health, Care, and Prevention (StMGPP), project “Zoonotic Bornavirus Focal Point Bavaria – ZooBoFo” (to B.S. and M.B.). In addition, this study was kindly supported by the intramural research ReForM-A program of the Faculty for Medicine of the University of Regensburg/University Hospital Regensburg (to M.B.).

## CRediT authorship contribution statement

**Lisa Arnold:** Writing – review & editing, Visualization, Validation, Methodology, Investigation, Formal analysis, Data curation, Conceptualization. **Vanessa Schwarz:** Investigation. **Gertrud Knoll:** Methodology, Investigation. **André Gessner:** Resources. **Barbara Schmidt:** Writing – review & editing, Resources, Methodology, Funding acquisition. **Markus Bauswein:** Writing – review & editing, Writing – original draft, Visualization, Validation, Supervision, Project administration, Methodology, Investigation, Funding acquisition, Formal analysis, Data curation, Conceptualization.

## Declaration of competing interest

The authors declare no competing interests related to this manuscript.

## Data Availability

Data will be made available on request.
