# Supplementary material for: Calu-3 cells as novel efficient cell culture system for Borna disease virus 1 (BoDV-1)
Source: Virus Res. 2026 Sep 10;373:199795. doi: 10.1016/j.virusres.2026.199795 (PMC13584004; doi:10.1016/j.virusres.2026.199795)
Supplement: Supplementary file 1 [file mmc1.docx]

**Supplement**

**Supplemental Figure S1**: Kinetics of BoDV-1 RNA shedding in supernatant were first examined using the BoDV-1 strain “Regensburg 2020” generated in Vero cells. Caco-2 and Calu-3 were infected (n = 6). Infection dynamics were slower compared to the BoDV-1 stock generated in Calu-3 cells (see Figure 1) due to the lower amounts of infectious particles contained in the stock generated in Vero cells. This experiment demonstrates that the phenotype of BoDV-1 RNA shedding in the supernatant of Calu-3 cells is not restricted to the virus stock generated in Calu-3 cells, thus excluding a cell-line-specific adaptation as underlying mechanism, although experimental conditions were less standardized in these initial experiments.

**Supplemental Figure S2**: Kinetics of BoDV-1 RNA shedding in supernatant of cell lines (A549, Calu-3, Vero) for different isolates of BoDV-1. Geometric means and geometric standard deviations are shown (n = 3). In brief, 10^4^ cells/well of the respective cell line were seeded in 24-well plates (Sarstedt, Nümbrecht, Germany) in a volume of 400 µL cell culture medium. On day 0, cell lines were infected with virus stocks of the BoDV-1 isolates “Regensburg 2019” (LR722643.1, cluster 1A) or “Regensburg 2022” (OR468968.1, cluster 2), containing the following BoDV-1 RNA concentrations: isolate 2019 6.8 x 10^5^/mL, isolate 2022 4.5 x 10^5^/mL. The BoDV-1 isolates had been obtained from brain tissue samples of patients by culture in Vero cells, before stocks were generated in Calu-3 cells. 100 µL of each stock were added to the cells, corresponding to the following BoDV-1 copy numbers per 10^4^ cells in a final volume of 500 µL: 6.8 x 10^4^ (isolate 2019), 4.5 x 10^4^ (isolate 2022). 24 h post infection (p.i.), cells were washed twice in D-PBS (Thermo Fisher Scientific). Medium was renewed and the volume per well was adjusted to 400 µL. On day 6 p.i., cells were detached with trypsin (PAN Biotech) and all cells were transferred in a 6-well plate (2 mL volume per well; Sarstedt) (indicated by the vertical dotted lines). Supernatants for BoDV-1 RT-qPCR were collected on day 0 (right after infection), day 3 p.i., day 6 p.i. (before detachment with trypsin), and day 10 p.i. BoDV-1 RNA copies were quantified by the BoDV-1 mix 1 RT-qPCR.

**Supplemental Figure S3**: (**A**) LDH activity in supernatants of Vero and Calu-3 cells permanently infected with BoDV-1 in comparison to uninfected cells (0 % control). In addition, 100 % controls (lysed permanently infected cells) are shown for consistency. (**B**) Calculated LDH release in permanently infected Vero and Calu-3 cells. Shown are means and standard deviations of one experiment with three replicates. The lactate dehydrogenase (LDH) release in Calu-3 and Vero cells permanently infected with BoDV-1 was measured using the Promega LDH-Glo Cytotoxicity Assay (Promega, Fitchburg, WI, USA) according to the manufacturer´s instructions. In brief, 10^4^ uninfected cells per well (0 % controls) and 10^4^ infected cells per well were seeded in 96-well plates. After two days of cultivation, supernatants were collected, centrifuged, and inactivated with 1 % Triton X-100 (Sigma-Aldrich/Merck, Burlington, MA, USA) (dilution 9:10). For measurement, supernatants were diluted 1:100 with LDH Storage Buffer. For 100 % controls, cell pellets of permanently infected cells were lysed with 1 % Triton X-100 for 15 min at 37 °C (dilution 9:10), centrifuged, and diluted 1:100 with LDH Storage Buffer. Absolute values of LDH activity in supernatant [mU/mL] were calculated fitting a standard curve with linear regression. For the calculation of LDH release [%], supernatants of uninfected cells served as 0 % controls, pellets of infected cells lysed with 1 % Triton X-100 served as 100 % controls. The following formula was applied:

$$LDH release [\%]=\frac{X-0 \% control}{100 \% control-0 \% control}\times100 \%$$

| **A549** | **Caco-2** | **Calu-3** |
| --- | --- | --- |
|  |  |  |
| **OL** | **SK-N-AS** | **Vero** |
|  |  |  |

**Supplemental Figure S4**: Immunostaining of cells 14 days p.i. Cells were harvested on day 14 p.i. A third of the cells was split in a 96-well microtiter plate (Ibidi, Gräfelfing, Germany) in a volume of 200 µL for immunofluorescence staining (in triplicates). Cells were incubated overnight at a humidified atmosphere (37 °C, 5 % CO_2_). After removal of the supernatant, plates were dried at room temperature for 2 h and fixed at 80 °C for 2 h, before storage at ─20 °C. A heat-inactivated well characterized human serum sample positive for anti-BoDV-1-IgG was added in a 1:2,560 dilution in TRIS buffer with Tween 20 (Sigma-Aldrich/Merck, Burlington, MA, USA) (pH 8.0). After incubation for 2 h, plates were washed three times with D-PBS (Thermo Fisher Scientific, Waltham, MA, USA) and incubated with a 1:200 dilution of a Cy-3-conjugated polyclonal rabbit anti-human-IgG antibody (Jackson ImmunoResearch, West Grove, PA, USA) for 2 h. After a final washing cycle, the assays were analyzed using fluorescence microscopy (BZ-9000; Keyence, Neu-Isenburg, Germany). A magnification of 20 was used. Cells with characteristic fluorescing spots in the nucleus were considered “infected”. Blue arrows indicate exemplary intranuclear spots.

| **Infected cells**  Anti-BoDV-1-IgG | **Infected cells**  DAPI | **Infected cells**  Overlay:  Anti-BoDV-1-IgG + DAPI | **Control (uninfected cells)**  Overlay:  Anti-BoDV-1-IgG + DAPI |
| --- | --- | --- | --- |
| A549 | | | |
|  |  |   infection rate app. 30 % |  |
| Caco-2 | | | |
|  |  |   infection rate < 10 % |  |
| Calu-3 | | | |
|  |  |   infection rate > 90 % |  |
| OL | | | |
|  |  |   infection rate > 90 % |  |
| SK-N-AS | | | |
|  |  |   infection rate app. 40 % |  |
| Vero | | | |
|  |  |   infection rate > 90 % |  |

**Supplemental Figure S5**: Immunostaining of cells generated in the infection kinetic experiment. Cells were harvested on day 14 p.i. and were frozen at ─80 °C. Thawed cells were taken into culture for another 12 days, before they were split in a 96-well microtiter plate in a volume of 200 µL for immunofluorescence staining. Cells were incubated in the microtiter plate at a humidified atmosphere (37 °C, 5 % CO_2_) for another two days. After removal of the supernatant, plates were dried at room temperature for 2 h and fixed at 80 °C for 2 h, before storage at ─20 °C. A heat-inactivated well characterized human serum sample positive for anti-BoDV-1-IgG was added in a 1:2,560 dilution in TRIS buffer with Tween 20 (pH 8.0). After incubation for 2 h, plates were washed three times with D-PBS and incubated with a 1:200 dilution of a Cy-3-conjugated polyclonal rabbit anti-human-IgG antibody for 1 h. In addition, cell nuclei were stained with DAPI (Roche, Basel, Switzerland) for 30 min. After a final washing cycle, the assays were analyzed using fluorescence microscopy (BZ-9000; Keyence, Neu-Isenburg, Germany). Cells were visualized using a magnification of 20. Exposure time for Cy3 signals was 1/2 s, exposure time for DAPI signals was 1/60 s. Cells with characteristic double staining of the nuclei were considered “infected”.

**Supplemental Figure S6**: MTT assay for the assessment of cell viability in the experiments with favipiravir. An MTT assay was performed to measure possible cytotoxicity in the experiments with favipiravir for Vero (n = 1) (**A**) and Calu-3 cells (n = 3; shown are the median and the 95% confidence interval) (**B**). Cells in 48-well plates 14 days after first application of favipiravir were subjected to the MTT assay. By discarding supernatants, the volume per well was adjusted to 100 µL, before 10 µL of 3-(4,5-dimethylthiazol-2-yl)-2,5-diphenyltetrazolium bromide (MTT; biomol, Hamburg, Germany) were added to each well. Plates were incubated for 1 h to allow for intracellular reduction of the soluble yellow MTT to the insoluble purple formazan dye. Subsequently, 100 µL of Lysing Buffer (10 % SDS in 10 mM HCl) were added to each well and plates were left overnight in an incubator at a humidified atmosphere (37 °C; 5 % CO_2_). The absorbance in each well was measured at a wavelength of 595 nm in a microplate reader (SpectraMax ABS Plus; Molecular Devices, San Jose, CA, USA). For each triplicate, the median was calculated and used for further analysis. The median of the controls without favipiravir was assigned a viability of 100 %. Percentages of viability of the corresponding favipiravir concentrations were calculated with respect to the control. Calculated values above 100 % were assigned a maximum value of 100 %.

**Supplemental Figure S7**: *De-novo* infection of Calu-3 cells in the presence of favipiravir. For the *de-novo* infection of Calu-3 cells, 7.5 x 10^3^ uninfected Calu-3 cells per well were seeded in 48-well plates (volume 200 µL) one day before infection (day ─1). On day 0, favipiravir was added in the following concentrations: 31.25 µM, 62.50 µM, 125 µM, 250 µM, 500 µM, 1,000 µM. Cells in medium only served as controls (0 µM favipiravir). Each concentration was tested in triplicate. The experiment was performed once. One hour after the application of favipiravir, cells were infected with an MOI of 0.05 with the BoDV-1 virus stock on day 0. Cells were washed 24 h after infection and medium with corresponding favipiravir concentrations was renewed (also on day 6 p.i. and on day 9 p.i.). Supernatants were harvested on day 6, 10, and 14 p.i. and were subjected to BoDV-1 RT-qPCR. (**A**) Individual values and geometric means are given. The dotted horizontal line marks the LLOD of the BoDV-1 RNA RT-qPCR (3.0 x 10^2^ copies/mL). For statistical analysis, a two-way repeated measures ANOVA with the Geisser-Greenhouse correction was performed, followed by Dunnett´s multiple comparisons test with respect to untreated cells (0 µM favipiravir). (**B**) Cell viability was assessed by an MTT assay 14 days p.i. The MTT assay was performed as described in the legend of Supplemental Figure S6. Values are expressed as percentages of the untreated control (0 µM favipiravir). Calculated values above 100 % were assigned a maximum value of 100 %.
